# Three discipline collaborative radiation therapy (3DCRT) special debate: Equipment development is stifling innovation in radiation oncology

**DOI:** 10.1002/acm2.12620

**Published:** 2019-05-25

**Authors:** Leonard Kim, Stephanie Markovina, Samantha J. Van Nest, Subarna Eisaman, Lakshmi Santanam, Julie M. Sullivan, Michael Dominello, Michael C. Joiner, Jay Burmeister

**Affiliations:** ^1^ Department of Radiation Oncology MD Anderson Cancer Center at Cooper Camden NJ USA; ^2^ Department of Radiation Oncology Washington University St. Louis MO USA; ^3^ Department of Radiation Oncology Weill Cornell Medical College New York NY USA; ^4^ Department of Radiation Oncology University of Pittsburgh Pittsburgh PA USA; ^5^ Department of Radiation Oncology Memorial Sloan Kettering Cancer Center New York NY USA; ^6^ Center for Devices and Radiological Health U.S. Food and Drug Administration Silver Spring MD USA; ^7^ Department of Oncology Wayne State University School of Medicine Detroit MI USA; ^8^ Gershenson Radiation Oncology Center Barbara Ann Karmanos Cancer Institute Detroit MI USA

## 1 THREE DISCIPLINE COLLABORATIVE RADIATION THERAPY (3DCRT) DEBATE

Radiation Oncology is a highly multidisciplinary medical specialty, drawing significantly from three scientific disciplines — medicine, physics, and biology. As a result, discussion of controversies or changes in practice within radiation oncology involves input from all three disciplines. For this reason, significant effort has been expended recently to foster collaborative multidisciplinary research in radiation oncology, with substantial demonstrated benefit 1, 2. In light of these results, we endeavor here to adopt this “team‐science” approach to the traditional debates featured in this journal. This article represents the fifth in a series of special debates entitled “three discipline collaborative radiation therapy (3DCRT)” in which each debate team will include a radiation oncologist, medical physicist, and radiobiologist. We hope that this format will not only be engaging for the readership but will also foster further collaboration in the science and clinical practice of radiation oncology.

## 2 INTRODUCTION

The field of radiation oncology has recently experienced a period of remarkable transformation of our technical capabilities. These technical and computational advancements have resulted in a tremendous improvement in our ability to precisely and accurately deliver radiation dose. However, this increased emphasis on technical capabilities may be inadvertently diverting attention and funding from scientific developments in other areas, even at a time when exciting new advances are occurring in cancer biology. Much of our recent technical development has been promoted and subsidized by the manufacturers of radiation oncology equipment. The question now becomes “who will encourage and subsidize the research needed to bring our recent biological advances to clinical fruition?” In a recent commentary which inspired this debate, Brown and Adler suggest that we “…are in a golden age of radiation and cancer biology” and that “…the industry's current focus on equipment development alone is undermining significant potential clinical advances in radiation oncology.”^3^ This is the subject of this month's 3DCRT debate.

Arguing for the proposition will be Drs. Leonard Kim, Stephanie Markovina, and Samantha Van Nest. Dr. Leonard Kim has worked on defining microscopic disease target volumes for breast cancer radiotherapy at William Beaumont Hospital and Rutgers Cancer Institute of New Jersey. He is currently Chief Medical Physicist at the MD Anderson Cancer Center at Cooper.

Dr. Stephanie Markovina is interested in improving therapeutic response to radiation in cervical cancer and other solid tumors by better understanding molecular mechanisms of radiation induced tumor cell death and survival. She is an Assistant Professor of Radiation Oncology and Cancer Biology at Washington University in St Louis where she specializes primarily in the treatment of patients with gynecologic cancers.

Dr. Samantha Van Nest has worked to establish spectroscopic techniques for the personalization of radiation therapy. She is currently a Postdoctoral Associate at Weill Cornell Medicine in New York, where her research investigates the effects of radiation on tumor immunity, with a particular focus on improving our understanding of the abscopal effect.

Arguing against the proposition will be Drs. Subarna Eisaman, Lakshmi Santanam, and Julie Sullivan. Dr. Subarna Hamid Eisaman is the clinical director and assistant professor at the University of Pittsburgh Medical Center (UPMC) Hillman Cancer Center Department of Radiation Oncology at the J. Murtha Pavilion in Johnstown, Pennsylvania. She serves as co‐chair of the Radiation Oncology Lung and Lymphoma Via Oncology Pathways Physician Advisory Committee. Her clinical practice includes treatment of breast, GYN, lung, CNS, head and neck, skin and musculoskeletal malignancies.

Dr. Lakshmi Santanam is an attending medical physicist at Memorial Sloan Kettering Cancer Center. Her primary interests include motion management and patient safety. She currently serves as vice chair for the AAPM Working Group on RO‐ILS and the Task Group on the Management of Respiratory Motion in Radiation Oncology.

Dr. Julie Sullivan is a biologist at the U.S. Food and Drug Administration's (FDA) Center for Devices and Radiological Health. Her scientific interests include the use of radiation‐emitting medical devices in clinical trials and the medical planning for and response to radiological and nuclear incidents.

## 3 OPENING STATEMENTS

### 3.1 Leonard Kim, MS, AMusD; Stephanie Markovina, MD, PhD; Samantha Van Nest, PhD

Radiation oncology is a science, and good science balances theory and experiment: the questions we seek to answer and the means to answer them. Without balance, progress and innovation can stall. For example, a “crisis” in high‐energy particle physics has been in the news lately, in which current technology does not or cannot validate theory.^4^ In radiation oncology, we have arguably the opposite imbalance. Equipment development is giving us answers to questions that perhaps should not be our top priority, questions such as “how good is my dose delivery now?” Certainly, continued improvement in delivery accuracy have contributed to the reduction in planning target volume (PTV) margins. But the clinical impact of further improvements to something that is already at submillimeter levels in some cases is certainly questionable given that, for example, the most sizable and uncertain margin today is probably the clinical target volume (CTV), which must be attacked through a better understanding of the biology underlying each cancer scenario rather than setup accuracy.

More insidiously, equipment development can influence the questions researchers choose to ask. This influence can take several forms. One is availability: though protons have been used clinically for decades, does it surprise anyone that there have been as many publications on proton radiotherapy in the past 5 years as in the 15 years previous to that? Characterizing newly available technology and its usage, even when it is not truly novel, is a comparatively easy path to the research productivity required for professional advancement in our field. It is a path additionally laden with incentives in the form of financial support from vendors. Although data are not generally available to the public, funding for research and development by these entities presumably favor efforts likely to generate new sources of revenue through either increased technical complexity, additional procedures, or expanded clinical indications. Given the continued cost to companies to develop and clinics to purchase up‐to‐date but mainstream technology (linear accelerators, high dose rate (HDR) remote afterloaders, imagers, etc.), development of existing equipment is likely the primary focus of research and development funding.

To be fair, many have found inspiration in asking, “what can I do with this new capability?” But it might be better if we did not let the tail of equipment development wag the dog of radiation oncology to the extent that it does. Forget capability for a moment. What are the questions we want to be answered, particularly clinical and biological questions, so that we can really innovate our field? Improved understanding of the biological effects of radiation as well as the biological factors that affect radiation response are needed. Exploring these fundamental processes could lead to the development of innovative new technologies that use radiation in ways we currently are not.

For example, a meta‐analysis of radiation therapy (RT) techniques for the treatment of medically inoperable early‐stage non‐small cell lung cancer (NSCLC) reported a 2‐year overall survival estimate of 53% for conventional RT.^5^ Improvements in radiation dose delivery and hypofractionation using stereotactic body radiation therapy (SBRT) improved 2‐year survival to 70%.^6^ Despite these improvements, distant disease recurrence develops in approximately 20% of patients following SBRT.^6^, ^7^ This is an example where clinical evidence suggests that despite technology‐based improvements in treatment delivery, there is an unmet need to address the systemic reduction of disease. Another example is the treatment of cervical cancer, where improved local control through advances in radiotherapy delivery such as image guidance for brachytherapy have not translated into improved cancer‐specific mortality, which for cervical cancer has not improved over the last several decades.^8^ Unpredictable systemic effects of radiation have not been adequately characterized or addressed. Both pro‐metastatic behavior as well as systemic reduction (known as the abscopal effect) have been observed in tumors treated with radiation.^9^, ^10^, ^11^, ^12^, ^13^, ^14^, ^15^, ^16^, ^17^ Radiation therapy and the technological drive toward conformality capitalize on the well‐characterized cytotoxic effect of radiation, but we need a better understanding of the dynamics between radiation and the complex biological system we are treating. This understanding in turn could lead to significant advances and even paradigm shifts in the way radiation is used to treat cancer. The use of immune modulation in combination with radiation therapy,^18^, ^19^, ^20^, ^21^ the characterization of microscopic disease that compose the CTV,^22^ the effects of tumor microenvironment,^23^ and genomic heterogeneity leading to potential differences in radioresistance^24^, ^25^ are all other important areas of research that have the potential to drastically change radiation oncology. By investing research efforts into a more fundamental understanding of the biological basis of our targets, we can potentially improve patient outcomes and better utilize the equipment that is already developed.

In the US, government funding accounts for much of the funding for radiation oncology research. While there appears to be an emphasis on cancer biology amongst National Institutes of Health (NIH) funded grants, the total funding for a treatment modality (radiation) which is applied to two‐thirds of all cancer patients is a mere 1.6% of NIH funding for cancer research.^26^ Additionally, radiation oncology vendors should be motivated to invest in better understanding of cancer biology and specifically radiation biology, particularly in the setting of newer techniques such as SBRT and the implementation of increasing numbers of targeted agents. Not only could the efficacy of traditional radiation therapy be maximized with this approach, but novel indications for radiation as discussed above could greatly expand the use of radiotherapy and thus existing radiotherapy equipment. New inquiry should also include the effect of different radiation modalities on “normal tissues” affected by other pathologies such as seizure disorder, cardiovascular disease, and cardiac conductivity disorders to name only a few.

In conclusion, the field of radiation oncology must prioritize overall improvement in the quality of life and effectiveness of our therapies as motivation for innovation and not be swayed by industry or economic pressures. Equipment development has no doubt allowed some of the biggest improvements in radiation therapy over the last several decades. However, this return is increasingly diminishing, and now is not the time to be continually tweaking already accurate, excellent equipment. Now is the time to devote resources and effort to answering pressing cancer and radiation biology questions. Any new equipment development should answer to these questions alone.

### 3.2 Subarna Eisaman, MD, PhD; Lakshmi Santanam, PhD; Julie Sullivan, PhD

Radiation oncology is unique and effective because it is a fusion of technology and biology with equipment development as an integral part of innovation. Often, equipment development pushes the boundaries of the field heralding a new era of novel therapies. For example, equipment development for delivery of intensity‐modulated radiation therapy (IMRT) supplanted older treatment techniques in many cancer sites, especially head and neck (H&N).^27^ Among many others, Wang et al.^28^ showed that better contralateral salivary gland sparing with IMRT improved patient saliva output and grade of xerostomia post treatment, a critical improvement in the patient's quality of life.

Furthermore, technological advances in image guidance and treatment delivery techniques paved the path for SBRT. SBRT provided an innovative way to target recurrent or second primaries within previously irradiated H&N cancers.^29^ A multi‐institutional study showed feasibility of loco‐regional control with SBRT in 197 H&N patients who had received previous median radiation dose of 70 Gy.^30^ Again, a huge step toward innovative solution for a patient population with extremely limited options. As Aznar et al.^31^ point out these equipment advances have paved the path to the first NRG Oncology Group initiated phase 1 clinical trial of SBRT for the treatment of multiple metastases in multiple organ sites (BR001; NCT02206334).^32^

Moving beyond 2 Gy fractions is unlikely to have occurred without knowing that treatment with higher doses could be done safely and effectively. The wide spread application of SBRT has in turn triggered innovative variations in radiobiological models. Universal survival curve (USC), published in 2008,^33^ compares different fractionations of both conventionally fractionated radiotherapy and SBRT. Mehta et al. suggest based on their clinical data for early stage NSCLC, the high rate of local tumor control achieved by SBRT can be fully explained by the much higher biological effective dose.^34^

With the development of high definition multi‐leaf collimators (MLCs), sophisticated treatment delivery systems, and image guidance, it is possible to precisely deliver very high doses of radiation. New ancillary systems like surface tracking, electromagnetic transponder tracking in addition to existing motion management systems paves the way to reduce tumor margins and potentially reduce normal tissue toxicity. The latest in the innovation chain are magnetic resonance (MR) guided radiotherapy systems that allow real‐time visualization of the tumor without any extra imaging dose to patients. This in turn helps overcome the common challenge of sparing uninvolved liver and nearby organs at risk (OAR) (e.g., kidney and bowel). Henke et al. 35 reported the first clinical outcomes of 26 patients treated for various liver malignancies showing MR‐guided SBRT is well tolerated and can provide excellent local control. This is being further fine‐tuned using daily online adaptive re‐optimization since significant inter‐fractional changes in OAR positions were observed despite breath‐hold stereotactic ablative radiation therapy delivery under MR‐guidance. For example, in 17 patients treated for adrenal metastases with total 84 fractions, online re‐optimization improved target coverage in 63% of fractions and reduced the number of fractions not meeting the V95% objective for gross tumor volume (GTV) and PTV.^36^

Outside of the external beam systems, equipment development has led to physical methods for pushing sensitive tissues away from tumors using rectal spacers intended to create a rectal‐prostate space, and HDR applicators specific to patient anatomy leading to a more conformal tumor dose. A phase III trial studying 222 men receiving 79.2 Gy in 1.8‐Gy fractions to the prostate demonstrated significant reduction in 3‐year incidence in rectal toxicity in favor of a hydrogel spacer (grade ≥ 2 toxicity 5.7% vs 0%; *P* = 0.012).^37^

The next major advances are likely to come from understanding how to best use focused‐radiation therapy to modulate a patient's biological response in combination with innovative radiation delivery equipment. Use of radiation to prime the immune system and enhance systemic responses to immunotherapy treatment is an active area of clinical research.^38^ Gene‐expression profiling and molecular imaging may allow for adjustment of radiation dose based on tumor radiosensitivity^39^ or response‐based adaptive therapy. Targeted therapies or nanoparticles could exploit tumor‐specific antigens, helping to further localize the effects of radiation, and increasing the potential to overcome cellular repair and hypoxia limitations. Kwatra et al. demonstrated 2.5‐fold increase in double stranded DNA breaks when plasmid DNA was bombarded with 60 KeV electrons in the presence of gold nanoparticles.^40^ Therefore, equipment development is not stifling innovation in radiation oncology, but rather facilitates high‐quality treatments that could lead to better clinical outcomes.

## 4 REBUTTAL

### 4.1 Leonard Kim, MS, AMusD; Stephanie Markovina, MD, PhD; Samantha Van Nest, PhD

The litany of equipment developments recited in the “against” opening statement have indeed made significant impacts on our field. However, these developments: IMRT, SBRT, image‐guided RT (IGRT), OAR‐sparing devices, all capitalize on the cytotoxic basis of RT: radiating and killing a region of interest. When referring to “the latest in the innovation chain,” MR‐guided radiotherapy systems, our opponents themselves essentially refer to this basis as “being further fine‐tuned,” hardly the language of true innovation. One of the main points of our opening statement is that equipment development in our field is overly focused on improved treatment delivery accuracy, dominating precious resources of time, and money for diminishing clinical gains. The way to maximize the benefit of even the most promising technologies (SBRT, MRI‐guided RT) is not to develop them further, but to understand how they work. For example, the biologic effective dose model does not explain why SBRT is less effective for colorectal cancer (CRC) metastases in the lung compared to NSCLC.^41^, ^42^ Nor can we determine how best to integrate SBRT techniques into systemic (metastatic) disease settings without better biological understanding.

Our esteemed opponents then list developments that many in the field of radiation oncology are excited about: the combination of radiation with immunotherapy, gene‐expression profiling and molecular imaging, and targeted therapies. But these areas depend less on further equipment development and absolutely require a better understanding of the biology of radiation and cancer. For example, one of the studies cited by our opponents (Ref. [38]) was done by colleagues at the current laboratory of one of our authors (SVN). Only a moderately increased radiation dose (6–9 Gy per fraction) was found to be necessary to stimulate immune activity, a dose already achievable by current clinical instrumentation.^43^ Rather than further equipment development, the key problem and limitation for any clinical implementation of the ideas proposed in the study (as well as the gene‐expression profiling study cited by our opponents (Ref. [39]) is biological validation and understanding, without which further funding approval, clinical trial initiation, and adoption by clinicians will be limited.

In the end, the conclusion to which our opponents arrive is our own exact point. The innovations in our field most likely to make a significant impact on patient care and outcomes will only be achieved through better understanding the biology of our therapies and how radiation treatment may be best applied in a biological context. Investing in these areas — and not equipment development — is exactly what our field should be doing starting no later than now.

### 4.2 Subarna Eisaman, MD, PhD; Lakshmi Santanam, PhD; Julie Sullivan, PhD

We agree with our colleagues' conclusion that radiation oncology must prioritize overall improvement in quality of life and effectiveness of therapies and that many gains are to be made by focusing on answering cancer and radiation biology questions. However, we want to highlight that equipment development IS part of innovation. Radiation oncology is a blend of biology, physics, and clinical care, and innovation in the field can only occur when equipment development occurs in parallel with understanding the biological basis behind the treatment. Often, equipment development outpaces our biological understanding as advances in technology have made engineering a device quicker than biological experimentation. Advances in molecular techniques such as gene‐expression profiling have been needed to further insights in general cancer biology. These in turn have opened new areas of investigation for radiation biology.^44^, ^45^, ^46^

While submillimeter accuracy may not be needed to treat all tumors, radiation therapy is used to treat a variety of tumor types that require different levels of accuracy and precision. One example of the huge clinical impact of submillimeter accuracy is during brain tumor radiation therapy, especially in children. Dose delivery in such cases is limited by the tolerance of normal tissues surrounding the target.^47^, ^48^ Even with the current precision, nearly all children undergoing brain tumor radiation therapy develop a certain level of cognitive deficits long‐term. The physical basis for the damage to the nontargeted brain cortex from MV x‐rays or Co‐60 gamma rays is the spatial distribution of the radiation they produce in the brain. Specifically, the doses produced to the brain tissue located proximal and distal to the target are excessive.^49^ Better accuracy translates to less normal brain cortex damage. Furthermore, without a reliable and safe way of accurately delivering radiation in the clinic, even the most detailed radiobiologic understanding would have limited translation into real patient care.

As for the concern regarding funding, we are in complete agreement that not enough NIH research dollars are allocated to the study of basic radiobiology and radiation oncology in general. However, until this can be changed, any additional funding to help move our field forward should be not be discounted. There are examples of equipment development based on evidence where private developers work in conjunction with the NIH to generate relevant clinical data. A good example is the emerging data for rectal sparing in prostate cancer. We know risk of rectal toxicity depends on the volume of the rectum that receives a high‐radiation dose. In a large prospective series, the percentage of rectum receiving > 70 Gy (V70) correlated with the occurrence of chronic rectal toxicity.^50^ Based on this information, in vitro work was supported by NIH grants and cadaveric studies funded by an equipment developing company to analyze risks, benefits, and dosimetric effects of prostate‐rectum separation using polyethylene‐glycol (PEG)‐based hydrogels.^51^ Evaluation in a prospective multicenter randomized controlled trial showed a significant reduction in late (3–15 months) rectal toxicity severity.^52^

Finally, our colleagues make the argument that we may have plateaued on accuracy due to CTV accounting for uncertain margins. Thus far SBRT ablative doses are delivered without a CTV, therefore, little to no room for uncertainty. As we head toward immune modulation in combination with radiation therapy and tumor microenvironment modulation with radiation, as predicted by our colleagues, we will likely need even more accuracy in radiation treatment delivery.

Overall, in order to deliver innovative radiation oncology treatments in the clinic, we will continue to require equipment development. Therefore, while it will be essential to answer cancer and radiation biology questions, equipment development will continue to be an integral part innovation in radiation oncology.

## CONFLICT OF INTEREST

None declared.
